# Identification of Potential Key Genes and Prognostic Biomarkers of Lung Cancer Based on Bioinformatics

**DOI:** 10.1155/2023/2152432

**Published:** 2023-01-18

**Authors:** Kaier Cai, Zhilong Xie, Yingao Liu, Junfeng Wu, Hao Song, Wang Liu, Xinyi Wang, Yinghuan Xiong, Siyuan Gan, Yanqin Sun

**Affiliations:** ^1^The Second Clinical Medical College, Guangdong Medical University, Dongguan, China; ^2^Department of Respiratory, The Second Affiliated Hospital of Guangdong Medical University, Zhanjiang, China; ^3^Department of Pathology, Guangdong Medical University, Dongguan, China; ^4^Biotissue Repository, The Affiliated Hospital of Guangdong Medical University, Zhanjiang, China

## Abstract

**Objective:**

To analyze and identify the core genes related to the expression and prognosis of lung cancer including lung adenocarcinoma (LUAD) and lung squamous cell carcinoma (LUSC) by bioinformatics technology, with the aim of providing a reference for clinical treatment.

**Methods:**

Five sets of gene chips, GSE7670, GSE151102, GSE33532, GSE43458, and GSE19804, were obtained from the Gene Expression Omnibus (GEO) database. After using GEO2R to analyze the differentially expressed genes (DEGs) between lung cancer and normal tissues online, the common DEGs of the five sets of chips were obtained using a Venn online tool and imported into the Database for Annotation, Visualization, and Integrated Discovery (DAVID) database for Gene Ontology (GO) enrichment and Kyoto Encyclopedia of Genes and Genomes (KEGG) pathway analyses. The protein–protein interaction (PPI) network was constructed by STRING online software for further study, and the core genes were determined by Cytoscape software and KEGG pathway enrichment analysis. The clustering heat map was drawn by Excel software to verify its accuracy. In addition, we used the University of Alabama at Birmingham Cancer (UALCAN) website to analyze the expression of core genes in P53 mutation status, confirmed the expression of crucial core genes in lung cancer tissues with Gene Expression Profiling Interactive Analysis (GEPIA) and GEPIA2 online software, and evaluated their prognostic value in lung cancer patients with the Kaplan–Meier online plotter tool.

**Results:**

CHEK1, CCNB1, CCNB2, and CDK1 were selected. The expression levels of these four genes in lung cancer tissues were significantly higher than those in normal tissues. Their increased expression was negatively correlated with lung cancer patients (including LUAD and LUSC) prognosis and survival rate.

**Conclusion:**

CHEK1, CCNB1, CCNB2, and CDK1 are the critical core genes of lung cancer and are highly expressed in lung cancer. They are negatively correlated with the prognosis of lung cancer patients (including LUAD and LUSC) and closely related to the formation and prediction of lung cancer. They are valuable predictors and may be predictive biomarkers of lung cancer.

## 1. Introduction

Cancer statistics in 2018 show that there were 18.1 million new cases of cancer around the world, of which lung cancer was the most frequently diagnosed (accounting for approximately 11.6% of the new cases). By reviewing relevant literature, it was found that whether smoking, whether there is a history of lung disease or a family history of cancer, whether there is a long-term exposure to air pollutants, and so on have an inevitable relationship with the occurrence of lung cancer [1]. Lung cancer has long been ranked among the top three cancers in terms of incidence rates and mortality globally, seriously threatening people's health and bringing a heavy economic burden to families and society [2]. The treatment methods for lung cancer mainly include surgery, chemoradiotherapy, and targeted drug therapy. With the development and innovation of precision medicine and the application of targeted drugs, the prognosis of lung cancer patients has been dramatically improved [3]. However, due to the complexity of lung cancer pathogenesis and the differences in individual genes, treatment targets still need further research.

The maturity of gene chip and bioinformatics analysis technology provides broader research space for cancer diagnosis and treatment. Bioinformatics technology has been widely used in scientific research. As we all know, drug-target (protein) interaction (DTI) is of great significance for research and development of new drugs and has great advantages for the pharmaceutical industry and patients. However, it is often expensive and time consuming to predict DTI using wet laboratory experimental methods. It has been found that the model PreDTI proposed based on machine learning method is obviously superior to other existing methods in predicting DTI. This model can be used to find new drugs for unknown diseases or infections, such as using existing drug compounds and SARS coronavirus 2 protein sequences to treat coronavirus [4].

At present, many researchers around the world are conducting research on COVID-19. According to literature reports, in order to find the sharing ways and drug targets of IPF patients infected with COVID-19, researchers use several innovative bioinformatics tools to design protein–protein interaction (PPI) networks, identify the interaction between TF gene and miRNA and common differentially expressed genes, and identify the activity of TF. We found some common associations that may lead to increased mortality in patients with SARS-CoV-2-infected IPF [5]. Other scholars used Gene Ontology and molecular pathway analysis to carry out functional analysis and found that IPF and COPD have some common links with the progress of COVID-19 infection. By applying computer structural biology and promoting immune information strategies, they developed a therapy based on immune epitopes. This research can recommend therapeutic compounds for IPF patients affected by SARS-CoV-2 virus [6–8].

Tumor has always been one of the difficult problems that scientists have overcome. In order to provide therapeutic targets for drug research and development of esophageal cancer, the author used gene expression analysis to identify molecular biomarkers. Using four different microarray datasets related to EsC from the comprehensive gene expression database, 1083 differentially expressed genes (DEGs) were identified, and 10 central genes were found from the PPI network. It was further found that the identified clusters were involved in biogenesis, ubiquitination and proteasome degradation, interleukin signal transduction, and Notch HLH transcription pathway [9]. Non-small-cell lung cancer (NSCLC) is a kind of high incidence malignant tumor. The author used microarray gene expression dataset GSE10245 to screen that stratifin may be a key biomarker of NSCLC and play a crucial role in the development of NSCLC [10]. In addition, in order to supplement the genetic research on the internal mechanism of polycystic ovary syndrome, the author identified the core genes involved in the pathogenesis of PCOS through bioinformatics analysis, identified four central genes (RARA, KPNB1, REL, and MAP1B) from the PPI network, and revealed important drug characteristics and potential therapeutic targets of PCOS [11].

The significance of this study is that we have been able to conduct the largest controlled and genetic study of lung cancer patients and normal people [12]. This study used bioinformatics to screen essential DEGs in lung cancer obtained from original microarray datasets from the GEO database. The key DEGs were analyzed and identified using the bioinformatics analysis method. Then, these DEGs were analyzed by DAVID software. After that, we use STRING database to analyze the PPI network of the obtained DEGs. And PPI networks were constructed and analyzed by Cytoscape software. The molecular complex detection (MCODE) technology has been very effective in performing the module analysis from the constructed PPI network. We use it to calculate the key core genes. After using multiple databases and online tools, the essential core genes expressing significant correlations with targeted therapy and prognosis of lung cancer (including LUAD and LUSC) were identified and verified. Through systematic analysis, genomic differences between normal people and lung cancer patients can be seen. According to the collection of GSE7670, GSE151102, GSE3353, GSE43458, and GSE1984 five datasets, DEGs were identified, and similar differentially expressed genes were screened from the total differentially expressed genes of the two groups of data. GO terms, cellular information pathways, and PPI network Cytoscape 3.6.1 were analyzed for the two datasets. According to the corresponding similar DEGs, the prognosis of lung cancer patients was predicted [12].

## 2. Materials and Methods

### 2.1. Data Sources

Using the GEO database in the National Center for Biotechnology Information (NCBI) (https://www.ncbi.nlm.nih.gov/), five chips, corresponding to GSE7670, GSE151102, GSE33532, GSE43458, and GSE19804, were screened by searching “lung cancer.” The GSE7670 dataset contained 35 lung cancer tissues (including LUAD and LUSC) and 31 normal tissues, the GSE151102 dataset contained 64 lung cancer tissues and 59 normal tissues, the GSE33532 dataset collected 80 lung cancer tissues and 20 normal tissues, the GSE43458 dataset contained 80 lung cancer tissues and 30 normal tissues, and the GSE19804 dataset included 60 lung cancer tissues and 60 normal tissues. These 5 GEO datasets contain lung cancer tissues and normal lung tissues with a large number of cases, respectively. By comparing the differential genes of cancer and adjacent tissues of these 5 GEO datasets and then taking their intersection, the data obtained are more representative and reliable.

### 2.2. Screening of Differentially Expressed Genes

GEO2R online analysis was carried out on the five groups of chip data obtained above. The screening conditions included *P* < 0.05 and |logFC| > 1. Those with logFC > 0 were regarded as the upregulated genes of the corresponding chip, and those with logFC < 0 were regarded as the downregulated genes of the corresponding chip. Then, the five groups of DEGs were obtained through a Venn online web tool (http://bioinformatics.psb.ugent.be/webtools/Venn/). A Wayne diagram was drawn to obtain the intersection of upregulated and downregulated DEGs in the five groups of chips.

### 2.3. GO Enrichment and KEGG Pathway Enrichment Analyses

We used the DAVID database (http://dabid.ncifcrf.gov/) for GO enrichment analysis. KEGG pathway enrichment analysis was performed on the intersection of DEGs obtained by Venn tool analysis to understand the biological processes and tumor-related pathways involved. *P* < 0.05 was taken as the inclusion standard.

### 2.4. PPI Network Construction and Screening of Essential Core Genes

Input the results including up- and downregulated genes obtained by DAVID into the online STRING database (http://string-db.org/) to get the relevant PPI network. Then, download the obtained network, and the obtained protein action network was imported into Cytoscape 3.6.1 (http://www.cytoscape.org/). Visualization used the MCODE plug-in in Cytoscape software to screen the PPI network modules and core genes between DEGs and then to select the most connected and closely related core genes through the built-in software. The screened core genes were entered into DAVID online software again and verified through KEGG pathway enrichment analysis. The critical genes in the core genes were selected, and the essential core genes were verified with Excel 2016 software to confirm the expression accuracy and scientific precision of the screening process.

### 2.5. Survival Analysis and Core Gene Expression

In UALCAN (http://ualcan.path.uab.edu/home/), the term essential core genes in the P53 mutation state was analyzed, and then, the GEPIA website was used (http://gepia.cancer-pku.cn/). The screened essential core genes of lung cancer were analyzed to verify the expression level of core genes in lung cancer tissues. In addition, use the GEPIA2 (http://gepia2.cancer-pku.cn/#correlation) to analyze the correlation of the four genes expressed in lung squamous cell carcinoma and lung adenocarcinoma. Finally, the Kaplan–Meier online plotter tool (http://kmplot.com/analysis/) was used to evaluate the core genes' predictive value in patients with lung cancer (including LUAD and LUSC).

## 3. Results

### 3.1. Screening of DEGs in Lung Cancer

**Figure 1:**
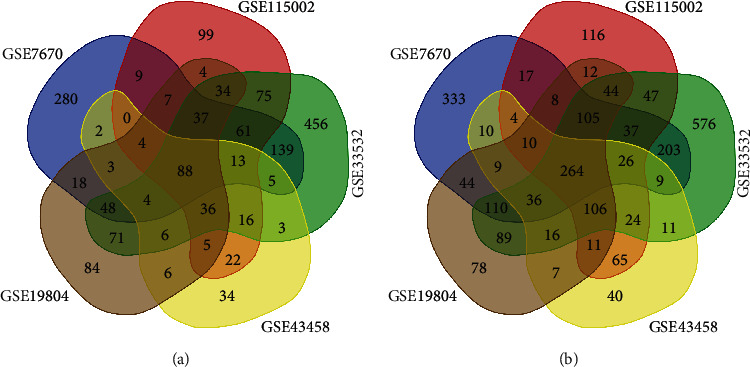
Venn intersection diagrams of the DEGs of the five datasets: (a) represents the upregulated gene expression, and (b) represents the downregulated genes.

Using the GEO2R online tool, we collected 2390, 1423, 3775, 955, and 1902 DEGs from GSE7670, GSE151102, GSE33532, GSE43458, and GSE19804. Then, an online Venn mapping tool was used to determine the intersection between lung cancer tissue and normal lung tissue. There were 352 DEGs (Supplementary documents Table S1), of which 88 genes (logFC > 1) were upregulated (Figure 1(a)), and 264 genes (logFC < −1) were downregulated (Figure 1(b)).

### 3.2. GO Enrichment and KEGG Pathway Enrichment Analyses of Lung Cancer DEGs

A total of 352 DEGs were subjected to GO analysis in DAVID online software (*P* < 0.05, Supplementary documents Table S2). The results showed that (1) in the biological process category (GOTERM_BP_DIRECT), the upregulated DEGs were particularly enriched in cell division, extracellular matrix tissue, cyclin-dependent protein serine/threonine kinase activity, G2/M transition of the mitotic cell cycle, collagen catabolism, and collagen fibril tissue; downregulated DEGs were particularly enriched in cell response to hormone stimulation, cell surface receptor signaling pathway, angiogenesis, cell adhesion, angiogenesis, and response to glucocorticoids. (2) In the process of cell composition (GOTERM_CC_DIRECT), the upregulated DEGs were particularly enriched in intermediates, spindles, cytoplasm, extracellular matrix, and centrosomes; downregulated DEGs were increased in the components of the plasma membrane, cell surface, receptor complex, membrane raft, and extracellular region. (3) In terms of molecular function (GOTERM_MF_DIRECT), the upregulated DEGs were particularly enriched in metal endopeptidase activity, microtubule binding, serine endopeptidase activity, microtubule motility, ATP binding, and collagen binding; downregulated DEGs in *β* matrix-binding protein and extracellular matrix-binding protein *β* were significantly enriched in binding, protein binding, and carbohydrate binding.

After KEGG pathway enrichment analysis, the results showed that (*P* < 0.05, Supplementary documents Table S3) the upregulated DEGs were mainly concentrated in the cell cycle, P53 signaling pathway, cell cycle yeast, cell aging, progesterone-mediated oocyte maturation, and yeast meiosis pathways; downregulated DEGs were mainly concentrated in the AGE-RAGE signaling pathway, PPAR signaling pathway, fluid shear stress and atherosclerosis, complement and coagulation pathways in malaria, vascular smooth muscle contraction, and diabetes complications.

### 3.3. PPI Analysis and Core Gene Screening

**Figure 2:**
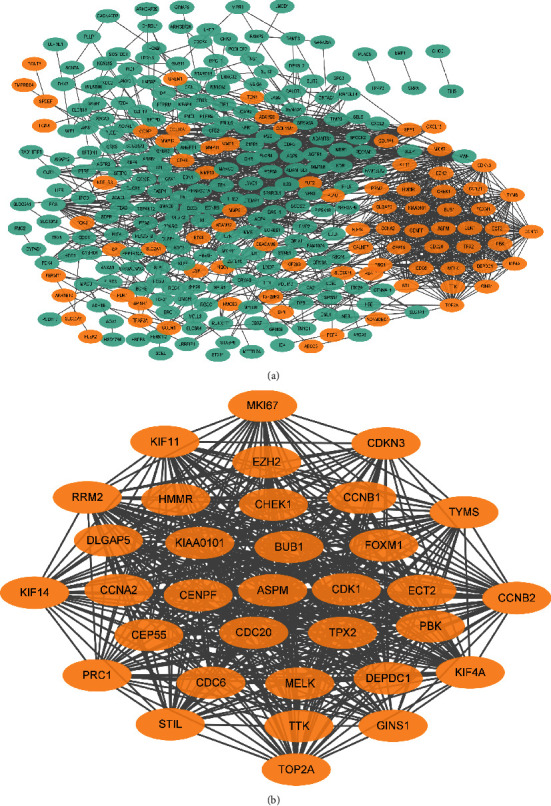
The common differentially expressed gene (DEG) protein–protein interaction network was constructed through a retrieval tool to retrieve the interacting genes and the core genes identified by the molecular complex detection (MCODE) application in Cytoscape. The orange circle indicates DEG upregulation, and the green circle indicates DEG downregulation: (a) shows 81 upregulated and 221 downregulated genes in the protein–protein interaction network, and (b) shows the core genes analyzed by MCODE analysis using Cytoscape software.

Using the online STRING database and Cytoscape 3.6.1, 352 DEGs were visualized after PPI network analysis (Figure 2(a)). A total of 352 DEGs displayed 302 nodes and 1454 edges in the PPI network, and 50 genes were not in the network. The MCODE plug-in in Cytoscape software was used to further analyze and verify the PPI network module and core genes of the DEGs. A total of 33 central nodes were most closely related during the period (Figure 2(b)), which contained 518 edges (see Table 1 for the details of the 33 genes).

We analyzed and screened the DAVID database to further narrow the scope and identify essential genes. We selected the cell cycle, P53 signaling pathway, and cellular senescence pathway (Table 2, Supplementary documents Figures S1–S3). Four genes were found to act on these three pathways simultaneously: CHEK1, CCNB1, CCNB2, and CDK1. Therefore, we hypothesized that these four genes are the critical core genes.

### 3.4. Accuracy and Reliability of Microarray Analysis of the Core Genes

**Figure 3:**
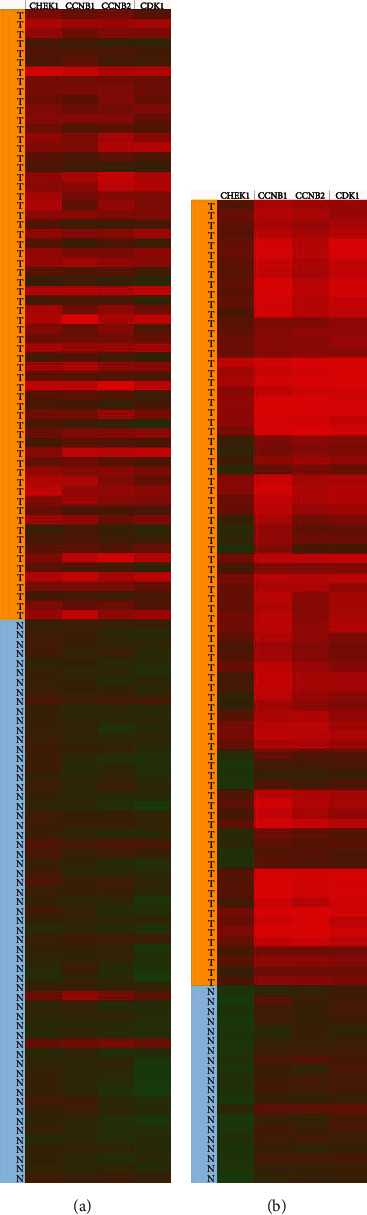
Validation and visualization of four essential core genes (CHEK1, CCNB1, CCNB2, and CDK1) in datasets GSE33532 and GSE151102. The heatmap was established based on the gene expression profiles in the information sets GSE151102 (a) and GSE33532 (b). The expression levels of DEGs are represented by different colors: red, high expression; green, low expression; blue, normal tissue; orange, lung cancer tissue.

The microarray datasets GSE7670, GSE151102, GSE33532, GSE43458, and GSE19804 were further analyzed to determine the accuracy and reliability of the related expression of the four essential core genes (CHEK1, CCNB1, CCNB2, and CDK1) in lung cancer. By analyzing the clustering heat maps of these four genes in the five chips produced by Excel 2016 software, significant differences in the expression of four essential core genes in normal tissues and cancer were noted (taking GSE151102 and GSE33532 as examples, as shown in Figure 3). The terms of these four critical core genes in lung cancer tissues were higher than those in normal tissues.

### 3.5. Relationship between the Expression of Core Genes in Lung Cancer-Related Information and the Prognosis and Survival of Lung Cancer Patients

**Figure 4:**
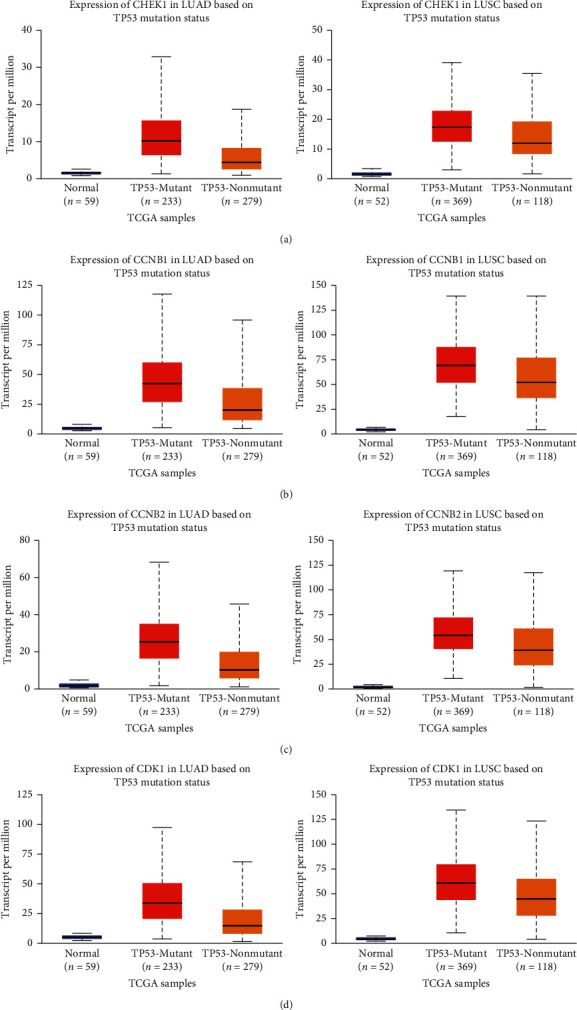
Expression of CHEK1 (a), CCNB1 (b), CCNBb2 (c), and CDK1 (d) in the P53 pathway mutation state on UALCAN.

UALCAN was used to analyze the expression of CHEK1, CCNB1, CCNB2, and CDK1 in the mutation state of the P53 pathway. The figure shows that the expression levels of the above core genes in normal tissues were significantly lower than those in tumor tissues without P53 mutation, and the expression levels of the above core genes in tumor tissues with P53 mutation were significantly higher than those in tumor tissues without P53 mutation (Figure 4, *P* < 0.05).

**Figure 5:**
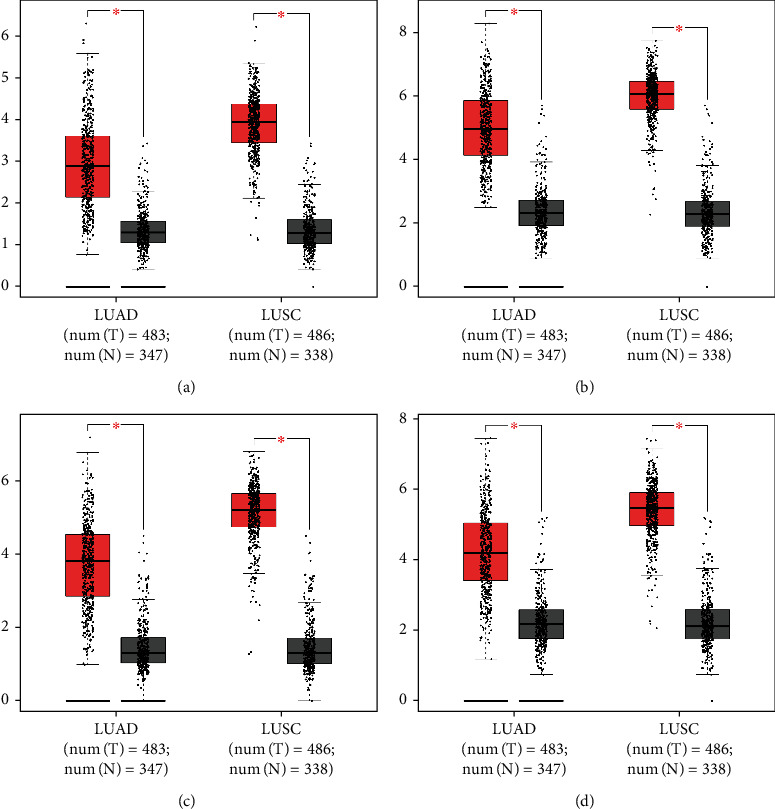
Expression of CHEK1 (a), CCNB1 (b), CCNB2 (c), and CDK1 (d) in lung cancer tissues compared to normal lung tissues.

GEPIA online software was used to analyze the expression of CHEK1, CCNB1, CCNB2, and CDK1 in 969 lung cancer tissues (including lung adenocarcinoma and lung squamous cell carcinoma) and 685 normal lung tissues. The expression levels of the four essential core genes in lung cancer tissues were significantly higher than those in normal tissues (Figure 5, *P* < 0.01).

**Figure 6:**
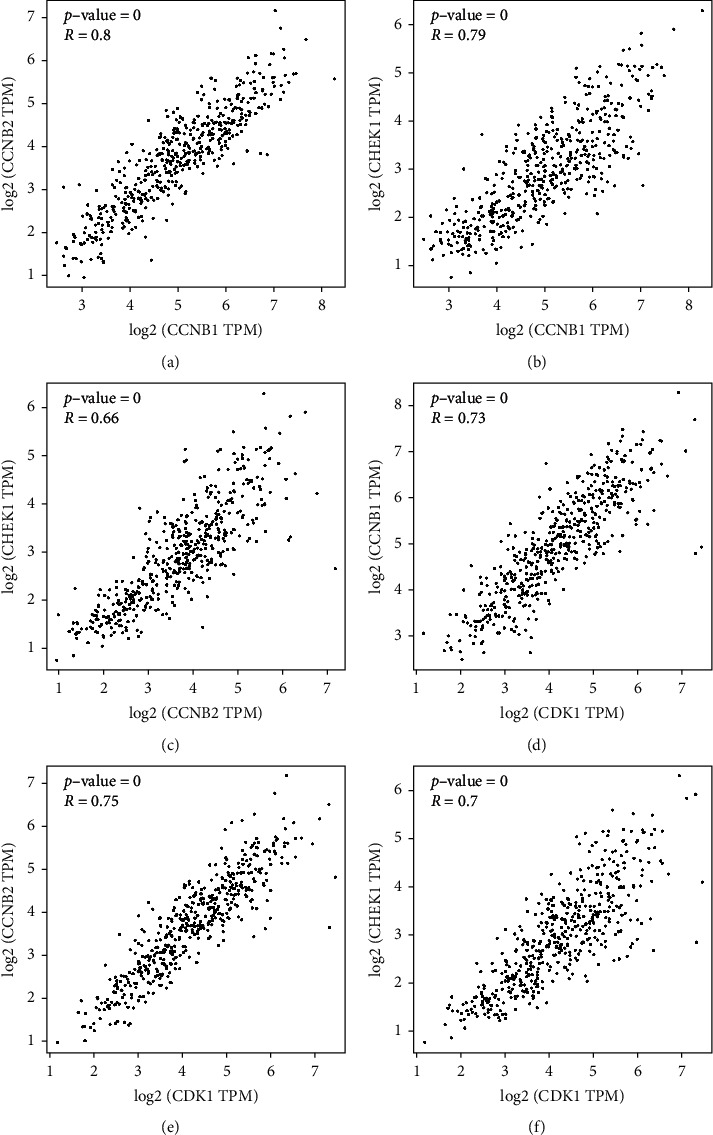
Correlation expression analysis of CHEK, CCNB1, CCNB2, and CDK1 in lung cancer tissues.

GEPIA2 online software was used to analyze the correlation of CHEK1, CCNB1, CCNB2 and CDK1 genes expressed in LUAD and LUSC patients. It can be found that the expression of CCNB1 and CCNB2 is highly correlated in lung cancer patients. The expressions of CCNB1 and CHEK1, CCNB1 and CDK1, CCNB2 and CHEK1, CCNB2 and CDK1, and CHEK1 and CDK1 are strongly correlated in LUAD and LUSC patients (Figure 6, *P* < 0.01).

**Figure 7:**
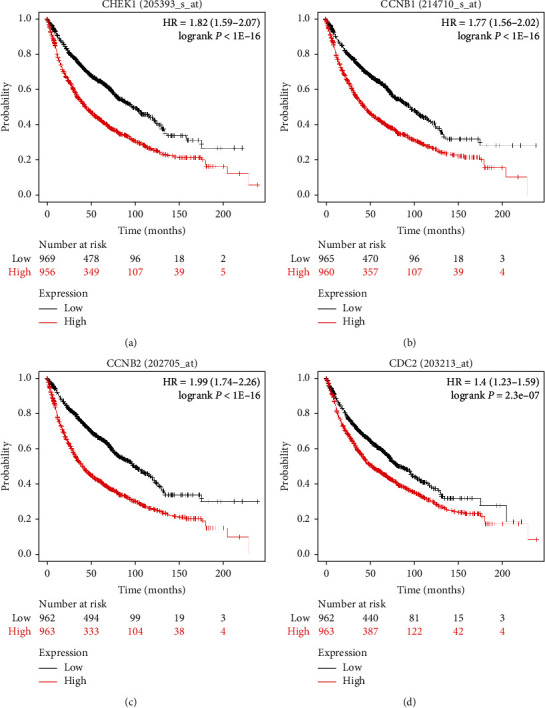
Kaplan–Meier correlation analysis on the prognosis of CHEK1, CCNB1, CCNB2, and CDK1 genes in patients with lung cancer.

Kaplan–Meier plotter was used to analyze the prognosis of the core genes CHEK1, CCNB1, CCNB2, and CDK1 in patients with lung cancer. The expression of CHEK1, CCNB1, CCNB2, and CDC2 was negatively correlated with the prognosis of patients with lung cancer (*P* < 0.01) (Figure 7).

## 4. Discussion

In this study, 352 DEGs were screened from the database, of which 88 genes were upregulated, and 264 genes were downregulated. After analyzing these DEGs, their enrichment pathways were divided into three groups: biological process, cell composition process, and molecular function. Further KEGG analysis revealed the main enrichment pathways of the DEGs. Then, using PPI network and Cytoscape software analyses, we obtained 33 core DEGs closely related to the central node. After enrichment analysis of the KEGG pathway for these 33 core DEGs, we found that four genes (CHEK1, CCNB1, CCNB2, and CDK1) were enriched in the cell cycle P53 signaling pathway and cellular senescence pathway at the same time (Figure 3). Therefore, we selected these four genes (CHEK1, CCNB1, CCNB2, and CDK1) as the critical core genes of this study.

Using the cluster heat map, UALAN website, GEPIA, and GEPIA2 online software and the Kaplan–Meier plotter online tool to analyze and verify the correlations of expression and survival rate of the four selected key core genes, it was found that the expression levels of these four genes in tumor tissues with P53 mutation were significantly higher than those in tumor tissues without P53 mutation and normal tissues. It is suggested that P53 mutation is highly correlated with these four genes and may play a coordinating role in the occurrence and development of tumors. At the same time, according to the analysis results of the above four pathways, the expression levels of CHEK1, CCNB1, CCNB2, and CDK1 in lung cancer tissues were significantly higher than those in normal tissues, and the expression of these four genes is negatively correlated with the prognosis and survival rate of lung cancer patients, and are valuable prognostic predictors.

The CHEK1 protein is a member of the Ser/Thr protein kinase family, which mediates cell cycle arrest by examining DNA replication and damage. Compared with healthy controls, the expression of the chek1 gene in lung cancer patients is relatively high [13, 14]. The term chek1 kinase is significantly correlated with TP53 mutation, which is highly expressed in cancer tissues and negatively associated with the patient's life cycle [15]. According to the above analysis results, when DNA damage occurs in the G2 phase of the cell cycle, chek1 will be phosphorylated and activated in an ATM-dependent manner and then initiate the TP53 pathway to arrest the cell cycle or perform further apoptosis, which is consistent with the literature report. In a study of TP53 mutant non-small-cell lung cancer tumor cells, it was found that inhibiting the expression of chek1 can significantly enhance the sensitivity of tumor cells to chemotherapy [16, 17]. In addition, promoter methylation, amplification, and miRNA regulation in patients with lung cancer may lead to the upregulation of the chek1 gene [18], which may be a marker for predicting the survival rate of patients with lung cancer [19]. It has also been reported that CHEK1 is associated with breast and gastric cancers and thus has important clinical and prognostic significance [20, 21].

Cyclin B1 (CCNB1) is a kind of regulatory protein that can promote cell division, metastasis, and cell differentiation. When DNA is damaged, the TP53 pathway activates and inhibits the binding of CDK1 and CCNB1 and induces apoptosis. In contrast, TP53 mutation can promote the formation of the CDK1-CCNB1 complex, accelerate the transformation of the cell cycle from G2 to the M phase, and induce lung cancer [22, 23]. Studies have shown that ccnb1 is generally highly expressed in tumor tissues. As a regulator of the cell cycle process, the expression of this protein in tumor cells is one of the indicators used to judge the degree of tumor malignancy [24–26]. This study shows that lung cancer patients with high expression of CCNB1 mRNA may have a poor prognosis, which can be used as an independent risk factor for poor prognosis in patients with lung squamous cell carcinoma.

CCNB2 is also a member of the cyclin family and may affect the proliferation, migration, and invasion of lung cancer cells by regulating the PI3K/Akt signaling pathway [27]. Roughly consistent with ccnb1, CCNB2 binds to CDK1 to form a complex and promotes G2/M transition by phosphorylating CDK1 kinase. During the G2/M transition, cells are inhibited and induce cell cycle arrest. The study found that CCNB2 was negatively correlated with the poor prognosis of lung cancer and was an independent predictor of poor prognosis in patients with lung adenocarcinoma; there was no significant difference in 5-year overall survival between patients with squamous cell carcinoma expressing lower and higher levels of CCNB2 mRNA [28, 29]. In addition, more research results show that the overexpression level of CCNB2 protein is significantly related to the degrees of tumor differentiation, tumor size, lymphatic metastasis, distant metastasis, and clinical stage [30, 31]. Therefore, CCNB2 is of great value in determining the prognosis of lung cancer and may become a potential target for lung cancer treatment.

Cyclin-dependent protein kinase (CDK) plays an important role in the G1/S and G2/M phases of eukaryotic cell cycle. Among them, the effect of CDK1 and cyclin A is in G2/M phase, while the combination of CDK1 and cyclin B plays a role in mitosis. According to the literature, as one of the core genes related to lung cancer, the activation of CDK can cause the phosphorylation of its target protein at the common site of CDK, thus promoting cell mitosis [32]. In the literature, the author used the cross analysis and follow-up study of tumor and normal tissues and used three datasets of differentially expressed genes. In contrast, our study used five datasets, based on gene expression in more cases. In the research result section, the differential expression of CDK1, CCNB1, and CCNB2 genes reported in the literature is consistent with this article, but we found four core genes in 969 lung tumor tissues and 685 normal lung tissues through in-depth analysis and verification, elaborated the expression of these four genes in lung squamous cell carcinoma and lung adenocarcinoma and their relationship with the prognosis of 1925 lung cancer patients, and analyzed their correlation with P53 mutation in lung cancer. Therefore, our conclusion is more scientific and reliable. This study shows that CDK1 is an important factor in cell cycle regulation. It plays a key role by stably binding with mitotic cyclin. Overexpression of CDK1 in lung cancer reduces chemosensitivity and is related to the lower survival rate of patients [33–35]. According to the literature, direct inhibition of CDK kinase activity is the basic strategy for developing effective cell cycle inhibitors [36]. Based on the results of this study, we speculate that CDK1 has excellent value in the survival and prognosis of lung cancer patients and may provide some possibilities for targeted drug delivery of lung cancer chemotherapy. Many studies have confirmed that CHEK1, CCNB1, CCNB2, and CDK1 may participate in lung cancer progression by affecting the cell cycle, DNA replication, homologous recombination, and the P53 signaling pathway [37]. Combined with the results of this study, it can be inferred that CHEK1, CCNB1, CCNB2, and CDK1 are critical genes involved in cell cycle arrest and DNA damage repair in lung cancer. Their abnormal regulation leads to chromosome abnormalities, uncontrolled cell proliferation, and apoptosis, forming malignant tumors. Therefore, CHEK1, CCNB1, CCNB2, and CDK1 may be helpful prognostic biomarkers for lung cancer. In the future, core genes may be used for the treatment and prognostic monitoring of lung cancer. The high expression of these four core genes in lung cancer patients can be used to indicate the prognosis of lung cancer patients and provide support for the diagnosis, treatment, and prognosis of lung cancer. In particular, for the targeted therapy of lung cancer, these four core genes may provide new directions for studying drugs for targeted therapies.

## 5. Conclusions

In this study, CHEK1, CCNB1, CCNB2, and CDK1 were screened and analyzed by comprehensive bioinformatics methods. The results show that these four genes play essential roles in the occurrence and development of lung cancer and are closely related to its prognosis and may become helpful prognostic biomarkers of lung cancer. However, further research and verification are needed.

## Figures and Tables

**Table 1:** Thirty-three core genes verified by MCODE plug-in analysis in Cytoscape software.

Gene name
CDKN3, KIF4A, BUB1, CEP55, CHEK1, STIL, RRM2, CDC6, DEPDC1, CDK1, ASPM, TTK, TYMS, TPX2, TOP2A, KIF11, CDC20, DLGAP5, CCNB2, CENPF, KIF14, HMMR, GINS1, MELK, MKI67, KIAA0101, CCNB1, PBK, ECT2, EZH2, CCNA2, FOXM1, and PRC1

**Table 2:** Results of KEGG pathway enrichment analysis of 33 core genes by DAVID (*P* < 0.05).

Pathway ID	Name	Count	%	*P* value	Genes
hsa04110	Cell cycle	8	29.63	0	CDC20, CCNB2, CCNB1, CHEK1, CDK1, TTK, CDC6, and BUB1
hsa04115	P53 signaling pathway	5	18.52	0	CCNB2, CCNB1, RRM2, CHEK1, and CDK1
Hsa04218	Cellular senescence	4	14.81	0.001	CCNB2, CCNB1, CHEK1, and CDK1

## Data Availability

The data used to support the findings of this study are available from the corresponding authors upon request.

## Supplementary Materials

Supplementary Materials Table S1: intersection table of DEGs from the five datasets obtained from the Venn diagram. Table S2: GO analysis of DEGs in lung cancer (*P* < 0.05). Table S3: results of KEGG pathway enrichment analysis of DEGs in lung cancer (*P* < 0.05). Figure S1: KEGG pathway enrichment analysis on 33 core genes (*P* < 0.05). CCNB2, CCNB1, CHEK1, and CDK1 were simultaneously enriched in the P53 signaling pathway. Figure S2: KEGG pathway enrichment analysis on 33 core genes (*P* < 0.05). CCNB2, CCNB1, CHEK1, and CDK1 were simultaneously enriched in the cell aging. Figure S3: KEGG pathway enrichment analysis on 33 core genes (*P* < 0.05). CCNB2, CCNB1, CHEK1, and CDK1 were simultaneously enriched in the cell cycle pathway.
