# Supplementary material for: Identification of Potential Key Genes and Prognostic Biomarkers of Lung Cancer Based on Bioinformatics
Source: Biomed Res Int. 2023 Jan 18;2023:2152432. doi: 10.1155/2023/2152432 (PMC9876670; doi:10.1155/2023/2152432)
Supplement: Supplementary Materials — Table S1: intersection table of DEGs from the five datasets obtained from the Venn diagram. Table S2: GO analysis of DEGs in lung cancer (P < 0.05). Table S3: results of KEGG pathway enrichment analysis of DEGs in lung cancer (P < 0.05). Figure S1: KEGG pathway enrichment analysis on 33 core genes (P < 0.05). CCNB2, CCNB1, CHEK1, and CDK1 were simultaneously enriched in the P53 signaling pathway. Figure S2: KEGG pathway enrichment analysis on 33 core genes (P < 0.05). CCNB2, CCNB1, CHEK1, and CDK1 were simultaneously enriched in the cell aging. Figure S3: KEGG pathway enrichment analysis on 33 core genes (P < 0.05). CCNB2, CCNB1, CHEK1, and CDK1 were simultaneously enriched in the cell cycle pathway. [file 2152432.f1.docx]

# Supplementary documents

Table 1 Intersection table of DEGs from the five datasets obtained from the Venn diagram

Table 2 GO analysis of DEGs in lung cancer (*P* < 0.05)

Table 3 Results of KEGG pathway enrichment analysis of DEGs in lung cancer (*P* < 0.05)

Figure 1: KEGG pathway enrichment analysis on 33 core genes (P < 0.05). CCNB2, CCNB1, CHEK1, and CDK1 were simultaneously enriched in the P53 signaling pathway.

Figure 2: KEGG pathway enrichment analysis on 33 core genes (P < 0.05). CCNB2, CCNB1, CHEK1, and CDK1 were simultaneously enriched in the cell aging.

Figure 3: KEGG pathway enrichment analysis on 33 core genes (P < 0.05). CCNB2, CCNB1, CHEK1, and CDK1 were simultaneously enriched in the cell cycle pathway.

Table 1. Intersection table of DEGs of five data sets obtained from Venn diagram

| **DEGS** | **Gene Name** |
| --- | --- |
| Up- regulated | KIF26B，SPINK1，ADAM12，PLAU，TPX2，MMP13，IGF2BP3，CCNB1，HMGB3，DSP，GOLM1，CXCL13，AKR1B10，GINS1，COL1A1，ADAMDEC1，FOXM1，EZH2，CDK1，CHEK1，KIF11，TOX3，CDC6，CST1，KIF14，KIF4A，ABCC3，SLC50A1，TMPRSS4，ETV4，SPP1，LGSN，TYMS，GALNT7，MELK，SFN，PLPP2，CDC20，SLC7A11，GREM1，CCNA2，KDELR3，BUB1，PBK，MMP1，MMP12，SLC2A1，ECT2，DEPDC1，MMP11，CDH3，ASPM，CRABP2，PLEK2，TCN1，STIL，NQO1，ADAM28，CCNB2，PRC1，CEACAM5，PSAT1，CEP55，SPDEF，RRM2，TOP2A，COMP，TFAP2A，DLGAP5，MKI67，PDK1，FERMT1，HMMR，SMPDL3B，GCNT3，PCP4，COL10A1，GPR87，COL11A1，FUT2，KIAA0101，LGR4，TTK，KCNN4，CDKN3，CP，CENPF，MMP9 |
| Down-，regulated | ABCA3，PLCE1，EDN1，ERG，AQP9，DKK2，PAPSS2，ANXA3，TEK，GRK5，VGLL3，GHR，MS4A2，EMP2，SLCO2A1，LAMP3，BCHE，STARD13，KL，PTRF，NPR1，SPTBN1，TSPAN7，ACADL，NEBL，HLF，NDNF，FHL1，KLF4，PPARGC1A，S100A3，ZFP36，TCF21，MEIS1，ARHGEF26，LDLR，SELP，SFTPD，GRIA1，OLR1，RUNX1T1，CD93，MSR1，WIF1，TPPP3，FLI1，RAMP3，CLIC5，PROS1，GIMAP6，FHL5，DOCK4，MME，LAMA3，ICAM2，PDK4，GPM6A，LRRFIP1，EFEMP1，P2RY14，LINC01140，WFS1，CACNA2D2，EMCN，ADARB1，LMO7，PPP1R15A，LRRC32，TSPAN12，NRN1，SASH1，GIPC2，OGN，SCGB1A1，CA4，PLCB4，PLA2G1B，TACC1，CXCL2，ABCA6，SH3BP5，LYVE1，P3H2，LEPROT///LEPR，PALMD，AKAP12，ARHGAP29，PDE5A，CPA3，CBX7，PID1，TBX3，SYNE1，ZBBX，PDZD2，LHFP，HBB，GNG11，SRPX，RAB11FIP1，PCDH17，SCN7A，FLRT3，SLC39A8，SFTPC，TGFBR3，LPL，PTPRM，SOSTDC1，FAXDC2，GPC3，LINC00312，CDH19，LMCD1，FOSB，MARCO，PIP5K1B，KIAA1462，EPAS1，SLIT2，CX3CR1，FZD4，PCOLCE2，CYBRD1，AGTR2，AOX1，HSD17B6，CYP4B1，SMAD6，ADGRE1，CHRDL1，HSPB8，ITIH5，SLC6A4，TMEM204，MMRN2，DPYSL2，ST6GALNAC5，ACVRL1，TIMP3，DACH1，EML1，TBX5，HBEGF，CRYAB，CD36，PTPRB，ADGRL2，AQP4，PPARG，MT1M，TNNC1，SELE，FAR2，CPB2，KLF6，THBD，EMP1，ABCA8，SLC19A3，AOC3，TMEM47，ADGRL4，ZBTB16，DES，ASPA，EDNRB，SLIT3，MYCT1，MYH10，STX11，MYH11，DUSP1，NPR3，SLPI，GPRC5A，RGCC，VWF，TIE1，ARRB1，DAPK2，VSIG4，AGTR1，TNS1，HPGD，SCEL，C7，WASF3，RAPGEF4，HEG1，IL6，ANGPT1，SEMA6A，TMEM100，CCDC68，DUOX1，ADGRG6，FERMT2，KDR，DPT，MFAP4，PECAM1，MAOB，FAM105A，ADGRB3，RBP4，CDO1，SDPR，ID4，ADAMTS1，ANOS1，SORBS1，LIFR，STXBP6，S1PR1，SLC1A1，PTPN21，FOS，LIMCH1，DENND3，SPOCK2，ADGRL3，LDB2，CALCRL，RECK，CAV1，CLEC1A，JAM2，SPARCL1，CA2，NXF3，METTL7A，ITM2A，FAM189A2，LRRN3，IL7R，FMO2，SOCS2，TGFBR2，IL33，TTN，PLLP，CTNNAL1，PGC，ADH1B，ARHGAP6，FABP4，FCN3，PDLIM3，CRTAC1，FAM107A，GPM6B，SEMA5A，RAMP2，MMRN1，VIPR1，PLAC8，KCNJ15，ADAMTSL3，CLDN18，CDH5，OLFML1，CNTN6，FBLN5 |

Table 2. GO analysis of DEGs in lung cancer (P < 0.05)

| **Expression** | **Category** | **Term** | **Count** | **%** | **P-Value** | **FDR** |
| --- | --- | --- | --- | --- | --- | --- |
| **Up-regulated** | GOTERM_BP_DIRECT | GO:0007052~mitotic spindle organization | 9 | 10.34482759 | 8.97E-08 | 4.80E-05 |
|  | GOTERM_BP_DIRECT | GO:0051301~cell division | 13 | 14.94252874 | 1.18E-07 | 4.80E-05 |
|  | GOTERM_BP_DIRECT | GO:0030198~extracellular matrix organization | 11 | 12.64367816 | 2.33E-07 | 6.34E-05 |
|  | GOTERM_BP_DIRECT | GO:0000079~regulation of cyclin-dependent protein serine/threonine kinase activity | 6 | 6.896551724 | 3.40E-06 | 6.93E-04 |
|  | GOTERM_BP_DIRECT | GO:0000086~G2/M transition of mitotic cell cycle | 7 | 8.045977011 | 3.77E-05 | 0.005342055 |
|  | GOTERM_BP_DIRECT | GO:0030574~collagen catabolic process | 5 | 5.747126437 | 3.93E-05 | 0.005342055 |
|  | GOTERM_CC_DIRECT | GO:0030496~midbody | 9 | 10.34482759 | 1.05E-06 | 1.4239904712570474E-4 |
|  | GOTERM_CC_DIRECT | GO:0005819~spindle | 8 | 9.195402299 | 2.75E-06 | 1.85E-04 |
|  | GOTERM_CC_DIRECT | GO:0000922~spindle pole | 7 | 8.045977011 | 1.7263780235284117E-5 | 7.77E-04 |
|  | GOTERM_CC_DIRECT | GO:0031012~extracellular matrix | 8 | 9.195402299 | 1.12E-04 | 0.003783036 |
|  | GOTERM_CC_DIRECT | GO:0005737~cytoplasm | 39 | 44.82758621 | 3.16E-04 | 0.008520306 |
|  | GOTERM_CC_DIRECT | GO:0005813~centrosome | 10 | 11.49425287 | 4.8232520199551816E-4 | 0.009327734 |
|  | GOTERM_MF_DIRECT | GO:0004222~metalloendopeptidase activity | 8 | 9.195402299 | 1.07E-06 | 2.42E-04 |
|  | GOTERM_MF_DIRECT | GO:0008017~microtubule binding | 8 | 9.195402299 | 1.95E-04 | 0.017437191 |
|  | GOTERM_MF_DIRECT | GO:0004252~serine-type endopeptidase activity | 7 | 8.045977011 | 2.3044745571999098E-4 | 0.017437191 |
|  | GOTERM_MF_DIRECT | GO:0004175~endopeptidase activity | 5 | 5.747126437 | 6.27E-04 | 0.035582462 |
|  | GOTERM_MF_DIRECT | GO:0003777~microtubule motor activity | 4 | 4.597701149 | 0.002546177 | 0.115596417 |
|  | GOTERM_MF_DIRECT | GO:0005518~collagen binding | 4 | 4.597701149 | 0.00348433704522778 | 0.131824085 |
| Down-regulated | GOTERM_BP_DIRECT | GO:0032870~cellular response to hormone stimulus | 9 | 3.488372093 | 2.43E-09 | 3.95E-06 |
|  | GOTERM_BP_DIRECT | GO:0007166~cell surface receptor signaling pathway | 21 | 8.139534884 | 4.00E-09 | 3.95E-06 |
|  | GOTERM_BP_DIRECT | GO:0001525~angiogenesis | 18 | 6.976744186 | 2.60E-08 | 1.71E-05 |
|  | GOTERM_BP_DIRECT | GO:0007155~cell adhesion | 25 | 9.689922481 | 2.45E-07 | 1.21E-04 |
|  | GOTERM_BP_DIRECT | GO:0001570~vasculogenesis | 9 | 3.488372093 | 1.32E-06 | 5.23E-04 |
|  | GOTERM_BP_DIRECT | GO:0051384~response to glucocorticoid | 9 | 3.488372093 | 2.15E-06 | 7.08E-04 |
|  | GOTERM_CC_DIRECT | GO:0005887~integral component of plasma membrane | 58 | 22.48062016 | 7.80E-15 | 2.33E-12 |
|  | GOTERM_CC_DIRECT | GO:0009986~cell surface | 36 | 13.95348837 | 1.12E-13 | 1.67E-11 |
|  | GOTERM_CC_DIRECT | GO:0005886~plasma membrane | 113 | 43.79844961 | 9.33E-12 | 9.30E-10 |
|  | GOTERM_CC_DIRECT | GO:0043235~receptor complex | 18 | 6.976744186 | 2.42E-09 | 1.81E-07 |
|  | GOTERM_CC_DIRECT | GO:0045121~membrane raft | 18 | 6.976744186 | 2.16E-08 | 1.25E-06 |
|  | GOTERM_CC_DIRECT | GO:0005576~extracellular region | 58 | 22.48062016 | 2.51E-08 | 1.25E-06 |
|  | GOTERM_MF_DIRECT | GO:0005509~calcium ion binding | 30 | 11.62790698 | 1.38E-07 | 6.99E-05 |
|  | GOTERM_MF_DIRECT | GO:0001540~beta-amyloid binding | 10 | 3.875968992 | 1.44E-06 | 3.65E-04 |
|  | GOTERM_MF_DIRECT | GO:0005515~protein binding | 190 | 73.64341085 | 1.41E-04 | 0.023798051 |
|  | GOTERM_MF_DIRECT | GO:0050431~transforming growth factor beta binding | 5 | 1.937984496 | 2.41E-04 | 0.030185714 |
|  | GOTERM_MF_DIRECT | GO:0030246~carbohydrate binding | 11 | 4.263565891 | 2.98E-04 | 0.030185714 |
|  | GOTERM_MF_DIRECT | GO:0008201~heparin binding | 10 | 3.875968992 | 4.28E-04 | 0.031660886 |

Notes：biological process (BP), molecular function (MF), cell component (CC).

Table 3 The analysis results of KEGG pathway enrichment of DEGs in lung cancer (P < 0.05)

| **Expression** | **Pathway ID** | **name** | **Count** | **%** | **PValue** | **Genes** |
| --- | --- | --- | --- | --- | --- | --- |
| Up- regulated | hsa04110 | Cell cycle | 10 | 11.49425287 | 2.68E-08 | CCNA2, CDC20, CCNB2, CCNB1, CHEK1, CDK1, TTK, SFN, CDC6, BUB1 |
|  | hsa04115 | p53 signaling pathway | 6 | 6.896551724 | 5.29E-05 | CCNB2, CCNB1, RRM2, CHEK1, CDK1, SFN |
|  | hsa04218 | Cellular senescence | 6 | 6.896551724 | 0.001790122 | CCNA2, CCNB2, CCNB1, CHEK1, CDK1, FOXM1 |
|  | hsa04914 | Progesterone-mediated oocyte maturation | 5 | 5.747126437 | 0.002564344 | CCNA2, CCNB2, CCNB1, CDK1, BUB1 |
|  | hsa04114 | Oocyte meiosis | 5 | 5.747126437 | 0.006275158120794178 | CDC20, CCNB2, CCNB1, CDK1, BUB1 |
|  | hsa04512 | ECM-receptor interactio | 4 | 4.597701149 | 0.013382618 | COL1A1, COMP, SPP1, HMMR |
| Down-  regulated | hsa04270 | Vascular smooth muscle contraction | 10 | 3.875968992 | 5.47E-04 | EDN1, RAMP2, PLCB4, CALCRL, RAMP3, NPR1, PLA2G1B, AGTR1, MYH11, MYH10 |
|  | hsa05144 | Malaria | 6 | 2.325581395 | 0.001723121 | SELP, IL6, PECAM1, HBB, CD36, SELE |
|  | hsa04933 | AGE-RAGE signaling pathway in diabetic complications | 8 | 3.100775194 | 0.00179243 | THBD, IL6, EDN1, PLCB4, AGTR1, PLCE1, SELE, TGFBR2 |
|  | hsa03320 | PPAR signaling pathway | 7 | 2.713178295 | 0.001933946 | FABP4, ACADL, LPL, OLR1, PPARG, SORBS1, CD36 |
|  | hsa05418 | Fluid shear stress and atherosclerosis | 9 | 3.488372093 | 0.002966617 | THBD, CDH5, EDN1, DUSP1, CAV1, PECAM1, KDR, FOS, SELE |
|  | hsa05202 | Transcriptional misregulation in cancer | 10 | 3.875968992 | 0.006454186 | IL6, MEIS1, HPGD, ZBTB16, TSPAN7, PPARG, ERG, FLI1, TGFBR2, RUNX1T1 |


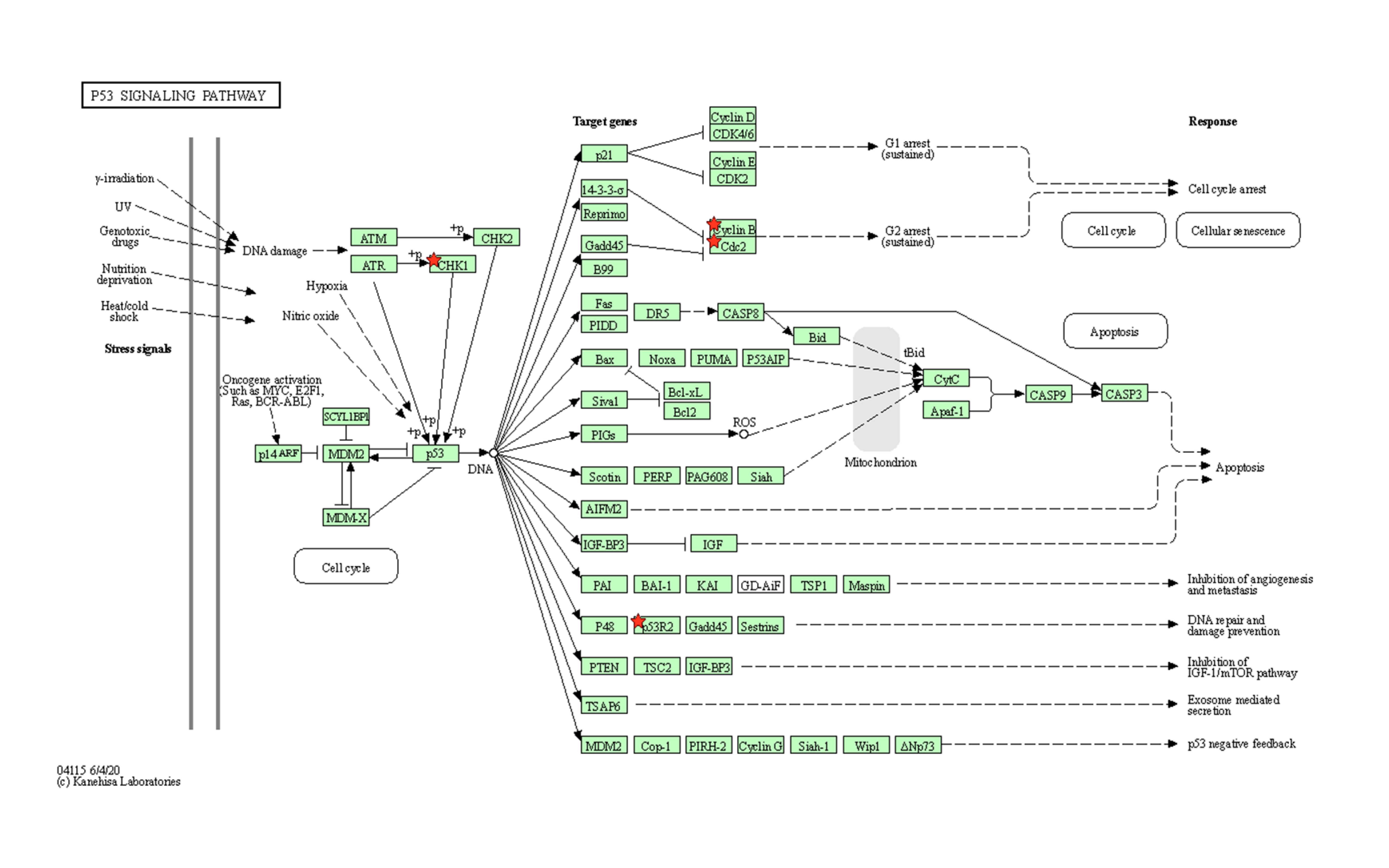


Fig. (1). KEGG pathway enrichment analysis on 33 core genes (P < 0.05). CCNB2, CCNB1, CHEK1, and CDK1 were simultaneously enriched in the P53 signaling pathway.


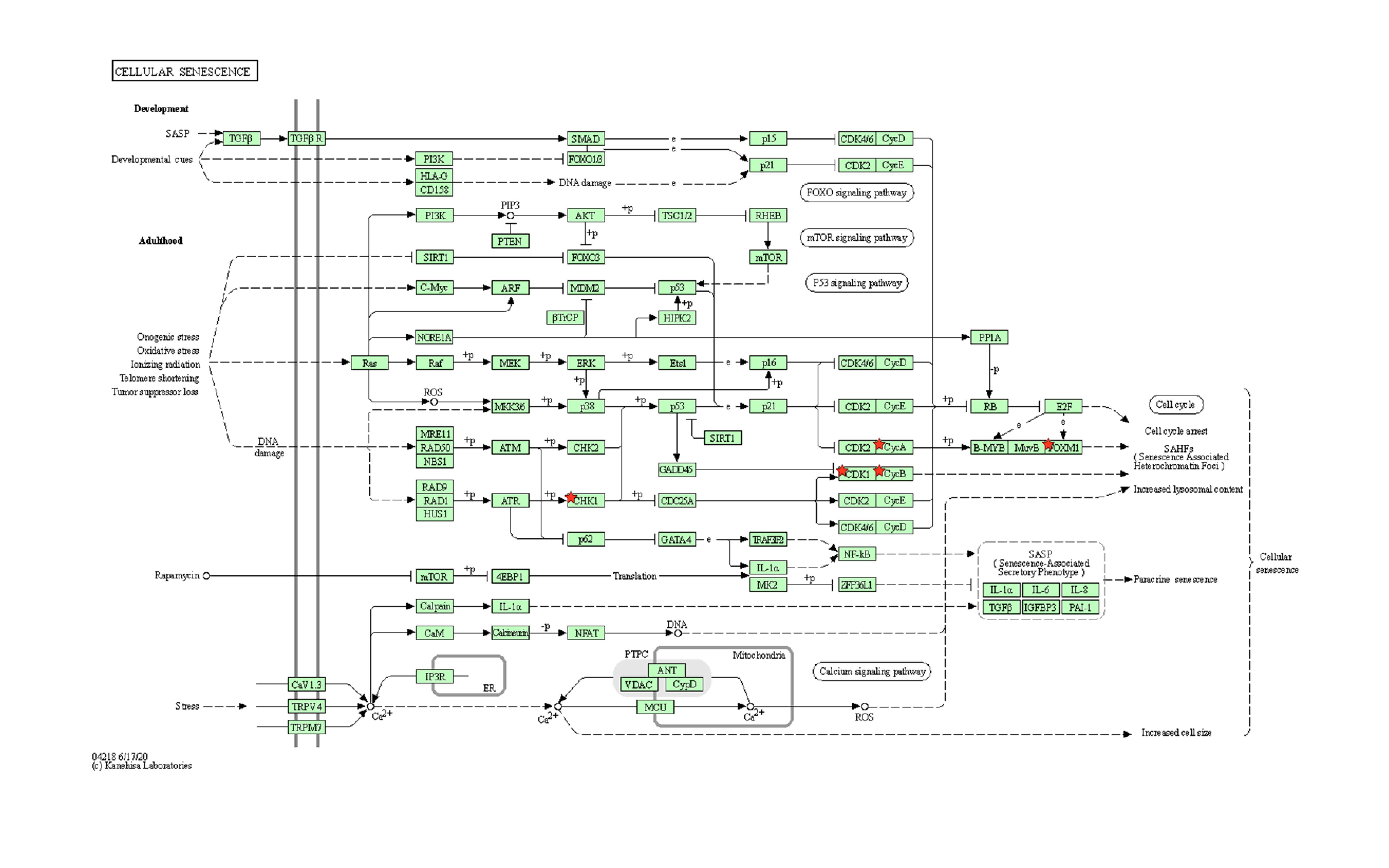


Fig. (2). KEGG pathway enrichment analysis on 33 core genes (P < 0.05). CCNB2, CCNB1, CHEK1, and CDK1 were simultaneously enriched in the cell aging.





Fig. (3). KEGG pathway enrichment analysis on 33 core genes (P < 0.05). CCNB2, CCNB1, CHEK1, and CDK1 were simultaneously enriched in the cell cycle pathway.
